# Supplementary material for: Promoting physical activity and academic achievement through physically active learning: Qualitative perspectives of co-design and implementation processes
Source: PLoS One. 2023 Nov 22;18(11):e0294422. doi: 10.1371/journal.pone.0294422 (PMC10664963; doi:10.1371/journal.pone.0294422)
Supplement: Attachment — Submitted filename: PONE-D-23-12396.docx [file pone.0294422.s002.docx]

The manuscript entitled: Promoting physical activity and academic achievement through physically active learning: qualitative perspectives of co-design and implementation processes describes teachers’ and students’ perspectives on the processes related to developing and implementing a curriculum designed to enhance movement and learning. The findings of this study will be useful and informative to the PLOS ONE audience however more background information is needed in the introduction and the methods as currently written is confusing. Below are my suggestions to improve the manuscript.

Major comments:

1. Introduction- It’s important to define for the reader what is meant by “physically active learning”. Movement integration into classrooms takes on many different forms (activity breaks vs. active learning) so it will be important to detail what time of movement integration you are referring to.
2. Introduction- it is unclear what the rationale for creating a new program is rather than using an existing evidence-based program. At the beginning of the Objectives section, the word “challenges” is used but is not fully described. Providing a clear rationale for the study is needed to support its publication.
3. Introduction- as a follow up to comment #2, greater discussion and a review of literature related to “challenges faced in implementing PAL interventions” is needed.
4. Introduction- in the section entitled “Theory”, three evidence-based movement integration programs are described but not theories. What is the theory of change that is being proposed that will influence teacher deliver and student engagement in the program. A diagram of a conceptual model informed by these programs would be very useful.
5. Methods- It is unclear how the teachers and students were selected to participate in this study. This is very important information to disclose for qualitative research.
6. Methods- A lot more detail is needed that describes the instruments used to assess the implementation of the PAL program. Descriptions of the teacher experimental notes, observation grid are needed. “Inadequate elements” of the program needs to be defined and the actual intervention needs to be described. Some of the information presented in the results section should be moved to the methods section. (example page 12, lines 195-203 and page 13, lines 215-226).
7. Methods- As a follow-up to comment 6, a study timeline would be incredibly useful to better understand the study design and protocols. As written it is unclear what happened when, and the ability to replicate the process described in this study is minimal.
8. Methods- Copies of all interview guides should be included as appendices.
9. Methods- the format for the teacher semi-structured interview and student focus groups needs to be described in more detail. On page 8, line 131, it states “the structure of the FG discussion followed the guidelines outlined by Morra-Imas”. These guidelines need to be briefly described here.
10. Methods- Please describe the implementation science framework that was used to inform your assessment of the implementation context and process. No framework is mentioned in the section titled: “implementation framework”.
11. Discussion- On page 26, line 498, the author mention that the PAL program added 10-12 minutes of MVPA in the classroom, but this data was not presented in the methods or results section. This information should be added to those section or presented in the Introduction if it has already been published in another manuscript.
12. Discussion- the study findings need to be contextualized within the three programs mentioned in the introduction section. Which components of Take 10!, Funterval, and Fit en Vaardig op school were included in this PAL program and did they elicit the same results.

Minor comments:

1. The abstract should disclose that this is a qualitative analysis. This information regarding the methods used in the study is missing.
2. The acronyms used in this manuscript severely reduced the readability of the content presented. 4GT, HPET, FG are not commonly used acronyms; please just use the terms teachers, students, and health/pe teachers. You can explain in the text that you are explicitly referring to fourth grade teachers and students.
