# Supplementary material for: Promoting physical activity and academic achievement through physically active learning: Qualitative perspectives of co-design and implementation processes
Source: PLoS One. 2023 Nov 22;18(11):e0294422. doi: 10.1371/journal.pone.0294422 (PMC10664963; doi:10.1371/journal.pone.0294422)
Supplement: Attachment — Submitted filename: Response to Reviewers.docx [file pone.0294422.s004.docx]

# **Response to Reviewers**

*Please note that line numbers may have changed due to having accepted previous revisions. The following modifications now have changes in tracking.*

**Reviewer 1: Comments to the Author**

The manuscript entitled: Promoting physical activity and academic achievement through physically active learning: qualitative perspectives of co-design and implementation processes describes teachers’ and students’ perspectives on the processes related to developing and implementing a curriculum designed to enhance movement and learning. The findings of this study will be useful and informative to the PLOS ONE audience however more background information is needed in the introduction and the methods as currently written is confusing. Below are my suggestions to improve the manuscript.

We appreciate your valuable feedback on our manuscript, and we acknowledge the need for additional background information in the introduction to provide a more comprehensive context for the study. Furthermore, we recognize that the methods section required clarification. We have carefully considered these suggestions and have made the necessary revisions to address these concerns. Please find below the modification made in response to each addressed comment.

**Major comments:**

1. Introduction- It’s important to define for the reader what is meant by “physically active learning”. Movement integration into classrooms takes on many different forms (activity breaks vs. active learning) so it will be important to detail what time of movement integration you are referring to.

Thank you for your comment. We have added a comprehensive section from lines 12 to 43 to better define the concept of a PAL program in the revised manuscript. This section provides a detailed explanation of the components and objectives of the PAL program implemented in this study.

To establish a connection between our developed program and existing empirical programs, we have included Table 1. This table presents the specific movement durations of the program developed in our research, allowing for valuable insights and comparisons with other empirical programs.

2. Introduction- it is unclear what the rationale for creating a new program is rather than using an existing evidence-based program. At the beginning of the Objectives section, the word “challenges” is used but is not fully described. Providing a clear rationale for the study is needed to support its publication.

We have addressed the issue by providing a clearer rationale for creating a new program in comparison to using an existing evidence-based program. Specifically, we have made modifications from lines 35 to 43 to elaborate on these issues. Additionally, we have revised the objective accordingly in section 1.2 to align it with the clarified rationale. These changes enhance the clarity and strengthen the overall argumentation of the manuscript.

3. Introduction- as a follow up to comment #2, greater discussion and a review of literature related to “challenges faced in implementing PAL interventions” is needed.

In the section where the issues are mentioned (lines 35 to 43), we have included a summary of the relevant literature that supports and contextualizes these challenges. This addition strengthens the manuscript by providing a comprehensive understanding of the existing literature on the topic. We appreciate your feedback, and these revisions contribute to the overall quality of the introduction.

4. Introduction- in the section entitled “Theory”, three evidence-based movement integration programs are described but not theories. What is the theory of change that is being proposed that will influence teacher deliver and student engagement in the program. A diagram of a conceptual model informed by these programs would be very useful.

We have taken your suggestion into consideration and made revisions to enhance the "Theory" section. Instead of simply describing the reference programs, we have included Table 1, which incorporates the main components from each of these programs and those included in the co-designed program aimed at facilitating classroom implementation and student engagement, providing a concise overview and facilitating comparison. Although we have not included a diagram of a conceptual model in this revision, we believe that the table adequately addresses the concerns raised. These modifications strengthen the theoretical framework of the study and improve the clarity of the manuscript.

5. Methods- It is unclear how the teachers and students were selected to participate in this study. This is very important information to disclose for qualitative research.

In response to your comment, we have provided clarification on the method used to select teachers and students in lines 222 to 237. This information now explicitly discloses the process of participant selection for the qualitative research conducted in this study. Thank you for bringing this to our attention.

6. Methods- A lot more detail is needed that describes the instruments used to assess the implementation of the PAL program. Descriptions of the teacher experimental notes, observation grid are needed. “Inadequate elements” of the program needs to be defined and the actual intervention needs to be described. Some of the information presented in the results section should be moved to the methods section. (example page 12, lines 195-203 and page 13, lines 215-226).

Thank you for your valuable suggestion. We have made significant improvements to address the need for more detailed descriptions of the instruments used to assess the implementation of the PAL program. In the revised manuscript, we have provided comprehensive and specific information on each tool. Furthermore, we have strengthened the justification for the selection of these instruments by incorporating references to relevant studies that have employed similar measures. Additionally, to enhance transparency and facilitate replication, we have included the actual instruments as appendices to the manuscript. These revisions ensure that readers have a clear understanding of the assessment methods employed in our study and can confidently evaluate the validity and reliability of the findings.

7. Methods- As a follow-up to comment 6, a study timeline would be incredibly useful to better understand the study design and protocols. As written it is unclear what happened when, and the ability to replicate the process described in this study is minimal.

Thank you for your suggestion. We have addressed the need for a study timeline in the revised manuscript. In lines 473 to 484, we have provided a detailed description of the study timeline, outlining the sequence of events and presenting a chronological overview of the research process. To enhance the visual representation of the timeline, we have also included Figure 1 and Figure 2, which visually depict the key milestones and activities in the implementation of the PAL program. These additions significantly improve the clarity of the study design and facilitate the replication of the research process.

8. Methods- Copies of all interview guides should be included as appendices.

As suggested, we have included the interview and focus group guidelines as appendices in the revised manuscript. The interview guide is now included as Appendix A, and the focus group guide is included as Appendix B. These appendices provide detailed information on the themes, sub-themes and questions used during the interviews and focus group sessions.

9. Methods- the format for the teacher semi-structured interview and student focus groups needs to be described in more detail. On page 8, line 131, it states “the structure of the FG discussion followed the guidelines outlined by Morra-Imas”. These guidelines need to be briefly described here.

As suggested, we have made the necessary revisions to provide a more detailed description of the teacher semi-structured interviews and student focus groups. Additionally, we have clarified that Table 3 summarizes the guidelines suggested by Morra-Imas, which were used to facilitate and guide the focus group sessions. These modifications have been made to enhance the transparency and comprehensibility of our methods.

10. Methods- Please describe the implementation science framework that was used to inform your assessment of the implementation context and process. No framework is mentioned in the section titled: “implementation framework”.

Thank you for your feedback. We have addressed your comment by adding the implementation science framework that was used to inform our assessment of the implementation context and process. In the revised manuscript, specifically in lines 524 to 546, we have described the scientific device that was utilized. Additionally, on lines 556 to 576, we have provided detailed information about the Cogni-Actif program. These additions provide a clearer understanding of the framework used to implement the PAL program in the classroom.

11. Discussion- On page 26, line 498, the author mention that the PAL program added 10-12 minutes of MVPA in the classroom, but this data was not presented in the methods or results section. This information should be added to those section or presented in the Introduction if it has already been published in another manuscript.

Thank you for your comment. We have taken your suggestion into consideration and have added information about the duration of moderate-to-vigorous physical activity (MVPA) in each session of the PAL program. This information has been included in the Theory section and in the description of the PAL program implementation.

12. Discussion- the study findings need to be contextualized within the three programs mentioned in the introduction section. Which components of Take 10!, Funterval, and Fit en Vaardig op school were included in this PAL program and did they elicit the same results.

Thank you for your suggestion. We have now included a comparison of our findings with the outcomes of the three reference programs (Take 10!, Funterval, and Fit en Vaardig op school) in the discussion section. This contextualizes our study within the existing literature and highlights the similarities between our PAL program and these programs. It emphasizes that incorporating physical activity into the school curriculum, as demonstrated by these programs, is not only feasible but also an innovative approach to reinforce academic content and promote increased physical activity levels among students.

**Minor comments:**

1. The abstract should disclose that this is a qualitative analysis. This information regarding the methods used in the study is missing.

As suggested, we added this information in the abstract.

2. The acronyms used in this manuscript severely reduced the readability of the content presented. 4GT, HPET, FG are not commonly used acronyms; please just use the terms teachers, students, and health/pe teachers. You can explain in the text that you are explicitly referring to fourth grade teachers and students.

We have made the suggested modifications in the manuscript to enhance its readability.

**Reviewer 2 : Comments to the Author**

1. As the term PAL was previously introduced, here you should use PAL instead of "physically active learning"

Thank you for your comment. We have revised the manuscript to consistently use the acronym PAL instead of the term "physically active learning" throughout the text after its first appartition..

2. As stated previously, this study aims to co-design a PAL program and assess its implementation in elementary schools. However, participants came only from 4th grade. Is there any possibility to widen the age range for the participants or should you limit the aspirations of implementation for your PAL program?

We have carefully considered your suggestion and have revised the objective of the study to align with the implementation that was carried out in this research. Furthermore, we have provided a detailed explanation in the manuscript (lines 245 to 257) to clarify the rationale behind selecting 4th-grade students as participants for this study.

3. I suggest including some key references linked to the use of these qualitative methods. This action will definitely support the foundations of the paper.

Thank you for your suggestion. In response, we have strengthened the foundations of our paper by including key references related to the use of qualitative methods. These references provide support for the selection of the qualitative method used in our study to assess the implementation of the PAL program.

4. I suggest including a process of member checking in your study. This will ensure inter-rater reliability, consisting of the participants checking and approving the results.

Thank you for your suggestion. We acknowledge that a formal process of member checking with the participants to ensure inter-rater reliability would have been beneficial for this study. However, due to practical limitations and constraints in accessing the students and teachers at the specific time when the data were analyzed, we were unable to implement a member checking process in this particular study. We appreciate your comment and take it into consideration for our future research endeavors.

5. I guess it should name figure 2 here.

Thank you for bringing this to our attention. You are correct that there was a mistake in referencing Figure 2 in the manuscript. We have addressed this issue in the revised version by making the necessary corrections and ensuring that the figure numbers are accurately attributed.

6. I guess these references should be included using the same format than in the rest of the paper (by using numbers).

We apologize for the inconsistency in the formatting of the references. In the revised version of the manuscript, we have ensured that all references are consistently formatted using numbers (Vancouver), as per the standard citation style used in the rest of the paper.
